# Long noncoding RNA SNHG1 silencing accelerates hepatocyte-like cell differentiation of bone marrow-derived mesenchymal stem cells to alleviate cirrhosis *via* the microRNA-15a/SMURF1/UVRAG axis

**DOI:** 10.1038/s41420-022-00850-8

**Published:** 2022-02-22

**Authors:** Jia Sun, Xuedong Sun, Sean Hu, Maoqiang Wang, Na Ma, Junhui Chen, Feng Duan

**Affiliations:** 1 Shenzhen Beike Biotechnology Research Institute, Shenzhen, 518057 P.R. China; 2 Intervention and Cell Therapy Center, Shenzhen Hospital of Peking University, Shenzhen, 518057 P. R. China; 3 Department of Interventional Radiology, the First Medical Center, Chinese PLA General Hospital, Beijing, 100853 P.R. China; 4 Department of Radiotherapy, the First Medical Center, Chinese PLA General Hospital, Beijing, 100853 P.R. China

**Keywords:** Cell biology, Diseases

## Abstract

Bone marrow-derived mesenchymal stem cells (BMSCs) can differentiate into hepatocyte-like cells (HLCs) to attenuate cirrhosis. Long noncoding RNA (lncRNA) SNHG1 has been demonstrated to orchestrate BMSC differentiation, whereas its role in cirrhosis remains elusive. Therefore, this study was performed to figure out whether lncRNA SNHG1 was involved in cirrhosis by affecting HLC differentiation of BMSCs. Mouse BMSCs were isolated, and the BMSC differentiation into HLCs was induced by hepatocyte growth factor (HGF). A cirrhotic mouse model was established using carbon tetrachloride and phenobarbital, followed by intravenous injection of BMSCs with manipulated expression of lncRNA SNHG1, microRNA (miR)-15a, and SMURF1. Subsequent to HGF induction, expression of hepatocyte-related genes, albumin secretion, and glycogen accumulation was increased in BMSCs, suggesting the differentiation of BMSCs into HLCs. Mechanistically, lncRNA SNHG1 bound to miR-15a that targeted SMURF1, and SMURF1 diminished ATG5 and Wnt5a expression by enhancing the ubiquitination of UVRAG. LncRNA SNHG1 or SMURF1 silencing or miR-15a overexpression promoted differentiation of BMSCs into HLCs and repressed cirrhosis of mice by upregulating ATG5 and Wnt5a *via* UVRAG. Conclusively, lncRNA SNHG1 silencing might facilitate HLC differentiation from mouse BMSCs and alleviate cirrhosis *via* the miR-15a/SMURF1/UVRAG/ATG5/Wnt5a axis.

## Introduction

Cirrhosis is the ultimate pathological outcome of numerous chronic liver diseases, of which fibrosis is the precursor [1]. Cirrhosis contributes to necroinflammation and fibrogenesis and possesses histological features of diffuse nodular regeneration surrounded by dense fibrotic septa and subsequent parenchymal regression and collapse of the hepatic architecture, which together result in marked distortion of the hepatic vascular architecture [2]. As reported, cirrhosis is attributed to alcoholism, nonalcoholic steatohepatitis, chronic hepatitis B virus, and hepatitis C virus infection [3]. Currently, there exist the major complications for cirrhosis, like renal and cardiac disturbances, ascites, gastroesophageal varices, and hepatic encephalopathy, which are mainly caused by portal hypertension and hyperdynamic circulation and their hemodynamic and metabolic influences [4]. Recently, bone marrow-derived mesenchymal stem cell (BMSC)-based therapy has emerged as an attractive treatment regimen for cirrhosis [5, 6]. Moreover, it has been widely documented that hepatocyte-like cells can be directly differentiated from BMSCs in vitro [7]. More importantly, hepatocyte-like cell (HLC) transplantation can be utilized as an effective therapy for cirrhosis [8]. Therefore, it is imperative to figure out the mechanism underlying the alleviating effects of differentiation of BMSCs into HLCs on cirrhosis.

As widely recognized, the involvement of long noncoding RNAs (lncRNAs) has been identified in liver fibrosis [9]. As a host to eight snoRNAs with 11 exons, lncRNA small nucleolar RNA host gene 1 (SNHG1) is located at 11q12.3 region of the chromosome, which is expressed in various types of tumors [10]. Moreover, SNHG1 has been documented to be implicated in liver disease, like liver cancer [11]. Besides, existing evidence has suggested the repressive role of SNHG1 upregulation in BMSC differentiation [12]. Intriguingly, it has been manifested that SNHG1 bound to microRNA (miR)-15a to protect against cardiomyocyte hypertrophy [13]. miR-15a loss can promote lung fibroblast activation to facilitate lung fibrosis in mice [13]. Also, miR-15a inhibition is able to cause promotion of hepatitis B virus-related hepatocellular carcinoma (HCC) [14]. Furthermore, it was predicted by bioinformatics analysis that there existed binding sites of miR-15a to 3'-untranslated region (UTR) of Smad ubiquitin regulatory factor 1 (SMURF1). SMURF1 downregulation triggered attenuation of renal interstitial fibrosis in kidney transplantation [15]. In addition, SMURF1 was capable of accelerating progression of liver cancer [16]. Notably, a prior work indicated that SMURF1 ubiquitinated UV radiation resistance-associated gene (UVRAG) to induce autophagosome maturation in hepatocellular carcinoma [17]. Intriguingly, UVRAG and autophagy-related gene (ATG) 5 are widely recognized key autophagy genes [18]. ATG5 was previously documented to assume a critical role in a positive feedback loop between Wnt signaling and autophagy in melanoma [19]. Wnt5a could potentiate HLC differentiation from human MSCs to improve liver function [20].

Given the aforementioned reports, we hypothesized that lncRNA SNHG1 might affect HLC differentiation from BMSCs through UVRAG by modulating miR-15a-targeted SMURF1. Therefore, our work was designed to figure out impacts of lncRNA SNHG1 on cirrhosis by orchestrating HLC differentiation of BMSCs via miR-15a/SMURF1/UVRAG/ATG5/Wnt5a axis.

## Results

### BMSCs were induced by hepatocyte growth factor (HGF) to differentiate into HLCs

**Fig. 1:**
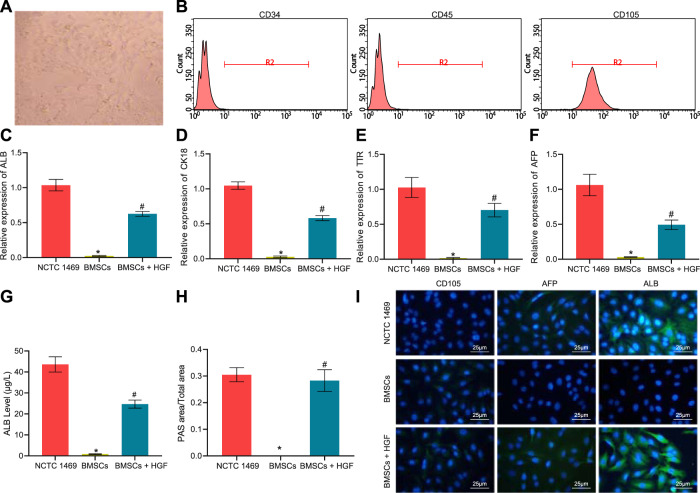
BMSCs are identified and induced to differentiate to HLCs by HGF. **A** Microscopic observation of cell morphology of BMSCs. **B** Flow cytometry analysis for CD105, CD34, and CD45 expression to identify BMSCs. **C** RT-qPCR to detect the expression of ALB in NCTC 1469 hepatocytes, HGF-treated BMSC, and untreated BMSCs. **D** RT-qPCR to detect the expression of CK18 in NCTC 1469 hepatocytes, HGF-treated BMSC, and untreated BMSCs. **E** RT-qPCR to detect the expression of TTR in NCTC 1469 hepatocytes, HGF-treated BMSC, and untreated BMSCs. **F** RT-qPCR to detect the expression of AFP in NCTC 1469 hepatocytes, HGF-treated BMSC, and untreated BMSCs. **G** ELISA to determine the content of ALB in the culture supernatant of NCTC 1469 hepatocytes, HGF-treated BMSC, and untreated BMSCs. **H** Glycogen content in NCTC 1469 hepatocytes, HGF-treated BMSC, and untreated BMSCs measured by PAS staining. **I** The expression of the BMSC marker CD105 and hepatocyte markers AFP and ALB in NCTC 1469 hepatocytes, HGF-treated BMSC, and untreated BMSCs, as detected with immunofluorescence staining (CD105/AFP/ALB in green, DAPI-stained nuclei in blue). *p < 0.05 *vs*. NCTC 1469 cells; ^#^*p* < 0.05 *vs*. untreated BMSCs. The cell experiments were repeated three times.

Initially, we isolated BMSCs from mice and observed the cellular morphology microscopically. Microscopic observation displayed that the cells were distributed in a monolayer and arranged radially (Fig. 1A). Flow cytometry revealed high expression of CD105, while almost no expression of CD34 and CD45 was observed in the cell population (Fig. 1B). The aforesaid results confirmed that the extracted cells were BMSCs.

Subsequently, the expression of hepatocyte-related markers (albumin [ALB], cytokeratin 18 [CK18], transthyretin [TTR], and alpha-fetoprotein [AFP]) in the BMSCs exposed to HGF was determined by reverse transcription quantitative polymerase chain reaction (RT-qPCR), with the hepatocyte cell line NCTC 1469 as a positive control. We found that BMSCs without HGF induction barely expressed ALB, CK18, TTR, and AFP, whereas these genes were expressed in both NCTC 1469 hepatocytes and HGF-challenged BMSCs (Fig. 1C–F). As reflected by enzyme-linked immunosorbent assay (ELISA), BMSCs supplemented with HGF had enhanced ALB secretion, whereas BMSCs without HGF barely secreted ALB, suggesting that they did not differentiate toward hepatocytes (Fig. 1G). Periodic acid-schiff (PAS) staining indicated that a larger amount of glycogen could be detected in hepatocytes and HGF-induced BMSCs, while BMSCs without HGF addition were negative for PAS staining (Fig. 1H). Additionally, the expression of the BMSC marker CD105 and hepatocyte markers AFP and ALB were analyzed using immunofluorescence staining. NCTC 1469 hepatocytes were observed to be CD105-negative yet AFP and ALB-positive; the presence of CD105 and the absence of AFP and ALB were identified in untreated BMSCs, whereas HGF-exposed BMSCs presented with markedly reduced CD105 expression yet increased AFP and ALB expression (Fig. 1I).

Collectively, BMSCs were successfully extracted and could differentiate towards HLCs by induction of HGF.

### Silencing lncRNA SNHG1 accelerated HLC differentiation of mouse BMSCs

**Fig. 2:**
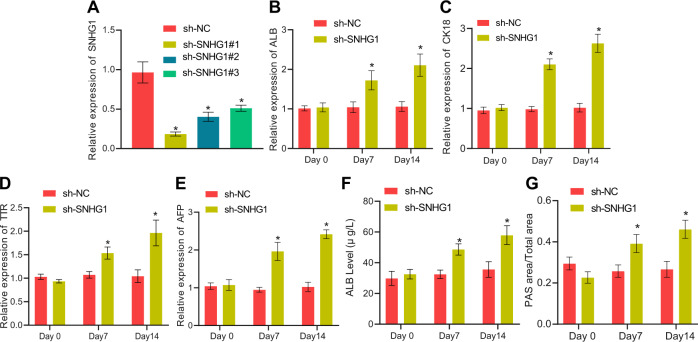
Silencing lncRNA SNHG1 promotes BMSCs to differentiate to HLCs. **A** Validation of lncRNA SNHG1 silencing efficiency by RT-qPCR. **B** RT-qPCR detection of the changes of ALB levels at day 0, 7, 14 after silencing of lncRNA SNHG1. **C** RT-qPCR detection of the changes of CK18 levels at day 0, 7, 14 after silencing of lncRNA SNHG1. **D** RT-qPCR detection of the changes of TTR levels after 0, 7, 14 days after silencing of lncRNA SNHG1. **E** RT-qPCR detection of the changes of AFP levels at day 0, 7, 14 after silencing of lncRNA SNHG1. **F** ELISA to detect the ALB content in the culture supernatant of BMSCs at day 0, 7, and 14 after silencing of lncRNA SNHG1. **G** Glycogen content in BMSCs at day 0, 7, and 14 after silencing of lncRNA SNHG1 determined by PAS staining. *p < 0.05 *vs*. BMSCs transfected with sh-NC. The cell experiments were repeated three times.

In order to investigate the effect of lncRNA SNHG1 on BMSCs, lncRNA SNHG1 was specifically silenced in BMSCs and the silencing efficiency was verified by RT-qPCR. The results documented that all three silencing sequences, especially sh-SNHG1#1, significantly reduced lncRNA SNHG1 expression (Fig. 2A). Therefore, the subsequent experimentation was implemented with sh-SNHG1#1. Moreover, lncRNA SNHG1 silencing resulted in significant increases in expression hepatocyte-related markers (Fig. 2B–E), ALB secretion (Fig. 2F), and glycogen content in the cells (Fig. 2G) at both 7 and 14 days. Conclusively, HLC differentiation of mouse BMSCs could be facilitated by silencing lncRNA SNHG1.

### Suppression of lncRNA SNHG1 in BMSCs alleviated cirrhosis in mice

**Fig. 3:**
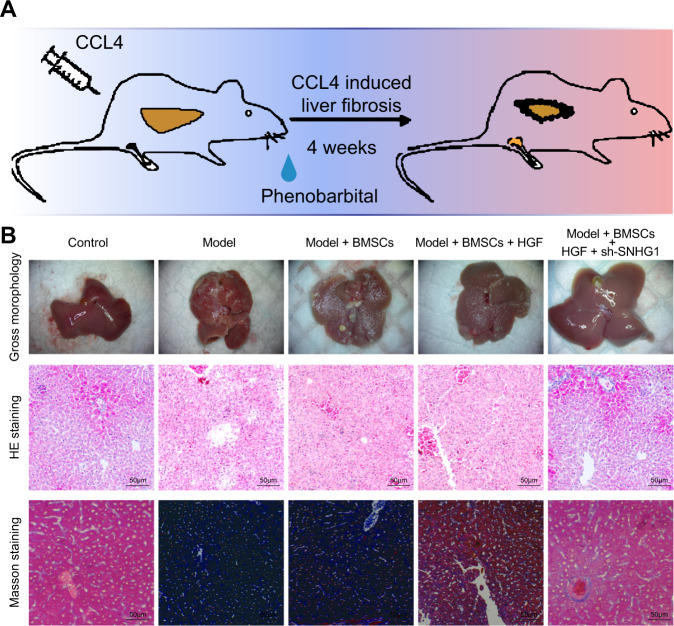
HGF-induced BMSCs silencing lncRNA SNHG1 attenuates cirrhosis in mice. **A** Schematic representation of mouse cirrhosis model establishment. **B** Liver gross observation, HE staining and Masson’s staining to detect liver morphology, liver cell necrosis, pseudolobule, and liver fibrosis in normal mice, cirrhotic mice, and treated mice. *N* = 6 mice/group.

The cirrhosis model was induced in mice to assess the role of lncRNA SNHG1 in treatment of BMSCs for cirrhosis. By simultaneously phenobarbital feeding and intraperitoneal injection of carbon tetrachloride for 4 weeks, we successfully developed a mouse cirrhosis model (Fig. 3A). The gross view displayed that compared with the liver of normal mice, the liver of cirrhotic mice was grayish-white, larger in size, and rough in surface (Fig. 3B), suggesting fibrosis formation. Furthermore, hematoxylin and eosin (HE) staining suggested necrosis of hepatocytes as well as regeneration of pseudolobules and Masson’s staining depicted obviously increased content of collagen fibers in the liver of cirrhotic mice relative to that of control mice, all of which indicated the successful establishment of cirrhosis. In addition, we observed little therapeutic effect on cirrhosis of tail vein injection of untreated BMSCs, whereas obvious alleviation of cirrhosis was witnessed after injection of HGF-induced BMSCs, while this effect was enhanced by silencing of lncRNA SNHG1 in the cells (Fig. 3B). The above findings were quantitatively confirmed by collagen fiber scoring (Supplementary Table 1). Taken together, BMSCs induced by HGF could effectively attenuate cirrhosis, and this effect could be further strengthened by silencing lncRNA SNHG1.

### LncRNA SNHG1 repressed HLC differentiation of BMSCs by regulating miR-15a/SMURF1 axis

Then, we studied the downstream mechanism of lncRNA SNHG1 in cirrhosis. LncRNA SNHG1 has been suggested to modulate miR-15a that may confer a promoting role in BMSC differentiation [13, 21]. SMURF1 has also been implicated BMSC differentiation [22], and was predicted to be a miR-15a target gene in our bioinformatics analysis. Thus, we speculated that lncRNA SNHG1 may mediate the potential miR-15a/SMURF1 cascade in HLC differentiation of BMSCs.

**Fig. 4:**
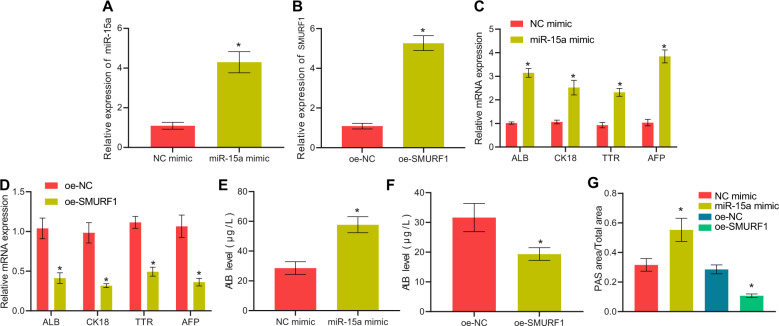
miR-15a and SMURF1 mediate HLC differentiation of BMSCs. **A** RT-qPCR validation of miR-15a mimic transfection efficiency in HGF-treated BMSCs. **B** RT-qPCR validation of oe-SMURF1 overexpression efficiency in HGF-treated BMSCs. **C** Changes in the levels of hepatocyte-related genes after overexpression of miR-15a for 14 days determined by RT-qPCR. **D** Changes in the levels of hepatocyte-related genes after overexpression of SMURF1 for 14 days measured by RT-qPCR, **E** RT-qPCR to assess the ALB content in the culture supernatant of BMSCs after overexpressing miR-15a for 14 days. **F** RT-qPCR to evaluate the secretion of ALB of BMSCs after overexpressing SMURF1 for 14 days; **G** Glycogen content in BMSCs after overexpressing miR-15a or SMURF1 for 14 days detected by PAS staining. *p < 0.05 *vs*. BMSCs transfected with oe-NC or NC mimic. The cell experiments were repeated three times.

HGF-treated BMSCs were transfected with miR-15a mimic or oe-SMURF1, followed by validation of the overexpression efficiency by RT-qPCR (Fig. 4A, B). As revealed in Fig. 4C–G, miR-15a mimic elevated hepatocyte-related gene expression, the secretion of ALB, and the accumulation of glycogen in BMSCs, whereas overexpression of SMURF1 led to the opposite results. Thus, miR-15a triggered yet SMURF1 attenuated HLC differentiation of BMSCs.

**Fig. 5:**
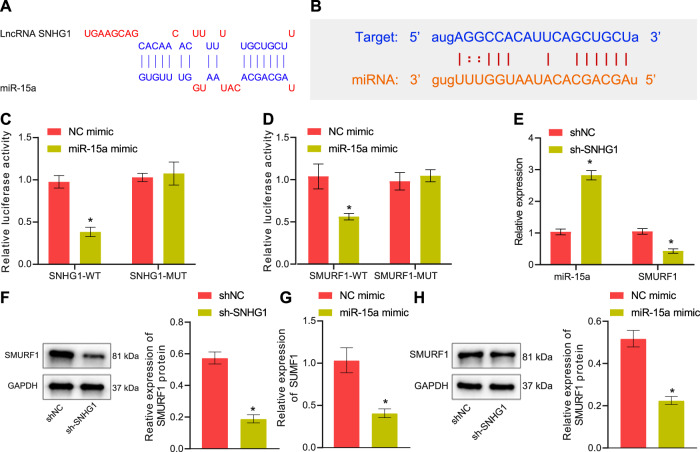
LncRNA SNHG1 binds to miR-15a to upregulate SMURF1. **A** Prediction of lncRNA SNHG1 binding sites with miR-15a and wild-type/mutant plasmid sequences. **B** Prediction of miR-15a binding sites in SMURF1 3'UTR and wild-type/mutant plasmid sequences. **C** Dual-luciferase assay to measure the binding of lncRNA SNHG1 to miR-15a. **D** Dual-luciferase assay to detect the binding of SMURF1 3'UTR to miR-15a. **E** The regulatory effect of lncRNA SNHG1 on SMURF1 and miR-15a determined by RT-qPCR. **F** The regulatory effect of lncRNA SNHG1 on SMURF1 and miR-15a detected by Western blot analysis. **G** RT-qPCR to assess the regulation of SMURF1 by miR-15a. **H** The regulation of SMURF1 by miR-15a detected by Western blot analysis. *p < 0.05 *vs*. BMSCs transfected with sh-NC or NC mimic. The cell experiments were repeated three times.

Further, the lncRNA SNHG1 binding sites on miR-15a and the miR-15a binding sites on SMURF1 3'UTR region were predicted (Fig. 5A, B) and the binding affinity among them was validated through dual-luciferase reporter assay (Fig. 5C, D). As indicated by RT-qPCR and Western blot analysis, lncRNA SNHG1 silencing led to the increased miR-15a expression and decreased SMURF1 expression (Fig. 5E, F), and that miR-15a mimic resulted in decline of SMURF1 expression (Fig. 5G, H). In summary, lncRNA SNHG1 may act as a miR-15a sponge to diminish miR-15a expression, and miR-15a may target and negatively regulate SMURF1.

**Fig. 6:**
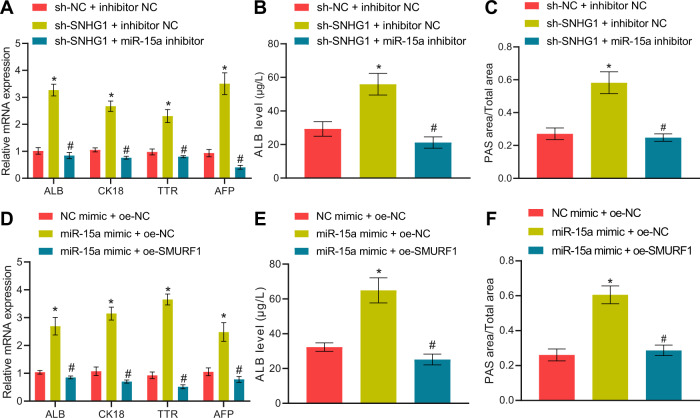
LncRNA SNHG1 silencing orchestrates the miR-15a/SMURF1 axis to induce HLC differentiation of mouse BMSCs. **A** RT-qPCR to detect hepatocyte-related gene expression in differentiated BMSCs after silencing of lncRNA SNHG1 or/and inhibition of miR-15a for 14 days. **B** ELISA to detect ALB content in culture supernatant of BMSCs after silencing of lncRNA SNHG1 or/and inhibition of miR-15a for 14 days. **C** The glycogen accumulation in BMSCs after silencing of lncRNA SNHG1 or/and inhibition of miR-15a for 14 days determined by PAS staining. **D** RT-qPCR to assess hepatocyte-related gene expression in differentiated BMSCs after overexpressing miR-15a or/and overexpressing SMURF1 for 14 days. **E** ELISA to assess ALB content in culture supernatant of BMSCs after overexpressing miR-15a or/and overexpressing SMURF1 for 14 days. **F** Glycogen accumulation in BMSCs after overexpression of miR-15a or/and overexpression of SMURF1 for 14 days evaluated by PAS staining. *p < 0.05 *vs*. BMSCs transfected with sh-NC + inhibitor NC/NC mimic + oe-NC; ^#^*p* < 0.05 *vs*. BMSCs transfected with sh-SNHG1 + inhibitor NC/miR-15a mimic + oe-NC. The cell experiments were repeated three times.

We next examined whether the lncRNA SNHG1/miR-15a/SMURF1 axis was involved in regulating HLC differentiation of BMSCs. First, silencing lncRNA SNHG1 in BMSCs resulted in notably augmented HLC differentiation degree of BMSCs, as reflected by increases in HLC marker expression, ALB secretion, and glycogen content, all of which were negated by additional treatment with miR-15a inhibitor (Fig. 6A–C). Meanwhile, miR-15a mimic enhanced HLC differentiation of BMSCs, whereas simultaneous overexpression of SMURF1 abrogated this effect (Fig. 6D–F).

In conclusion, lncRNA SNHG1 restricted HLC differentiation of mouse BMSCs through sponging miR-15a and thus upregulating SMURF1 expression.

### LncRNA SNHG1 silencing mediates the miR-15a/SMURF1 axis to alleviate cirrhosis in mice

Next, our focus was shifted to the role of the lncRNA SNHG1/miR-15a/SMURF1 axis in cirrhosis in mice. HGF-induced BMSCs with manipulated SNHG1/miR-15a/SMURF1 expression, after 7-d culture in vitro, were injected into mice to observe their effects on cirrhosis in mice.

The expression of lncRNA SNHG1, miR-15a, and SMURF1 was first determined with qRT-PCR in dissected mouse livers. Relative to Control mice, cirrhotic mice presented with upregulated SNHG1 and SMURF1 yet downregulated miR-15a; injection of sh-SNHG1-treated, HGF-induced BMSCs led to reduced SNHG1 and SMURF1 expression and elevated miR-15a expression in livers of cirrhotic mice, and, in contrast, injection of cells simultaneously silenced SNHG1 and miR-15a resulted in reduced miR-15a expression and enhanced SMURF1 expression, and had no additional influence on the SNHG1 expression (Supplementary Fig. 1A). In parallel, unaffected SNHG1 expression, upregulated miR-15a expression, and downregulated SMURF1 expression were observed in livers of cirrhotic mice injected with HGF-stimulated BMSCs overexpressing miR-15a; compared to that, simultaneous overexpression of SMURF1 in the cells elevated only SMURF1 expression and had no effects on the others (Supplementary Fig. 1B).

**Fig. 7:**
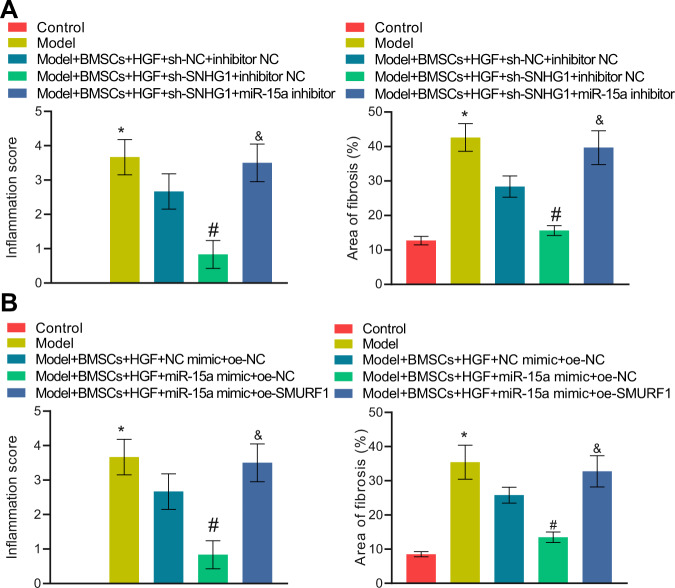
LncRNA SNHG1 silencing attenuates cirrhosis in mice via the miR-15a/SMURF1 axis. **A** HE staining and Masson’s staining results of normal mice, cirrhotic mice, and mice treated with sh-lncRNA SNHG1 alone or in combination with miR-15a inhibitor. **B** HE staining and Masson’s staining results of normal mice, cirrhotic mice, and mice treated with miR-15a mimic alone or in combination with oe-SMURF1. *N* = 6 mice/group. *p < 0.05 *vs*. the Control group; ^#^*p* < 0.05 *vs*. the Model + BMSCs + HGF + sh-NC + inhibitor NC or the Model + BMSCs + HGF + mimic NC + oe-NC group; and *p* < 0.05 *vs*. the Model + BMSCs + HGF + sh-SNHG1 + inhibitor NC or the Model + BMSCs + HGF + miR-15a mimic + oe-NC group.

Silencing lncRNA SNHG1 alone in BMSCs remarkably ameliorated cirrhosis in mice, whereas its combination with miR-15a inhibition reversed such therapeutic effect (Fig. 7A, Supplementary Table 2). Consistently, miR-15a mimic treatment in BMSCs noticeably restrained cirrhosis in mice, whilst simultaneous overexpression of SMURF1 nullified the effect of miR-15a restoration alone (Fig. 7B, Supplementary Table 3).

These results suggested that lncRNA SNHG1 protected against cirrhosis in mice through the lncRNA SNHG1/miR-15a/SMURF1 axis.

### SMURF1 promotes ubiquitination of UVRAG and inactivates ATG5/Wnt5a to inhibit HLC differentiation of mouse BMSCs and facilitate cirrhosis in mice

**Fig. 8:**
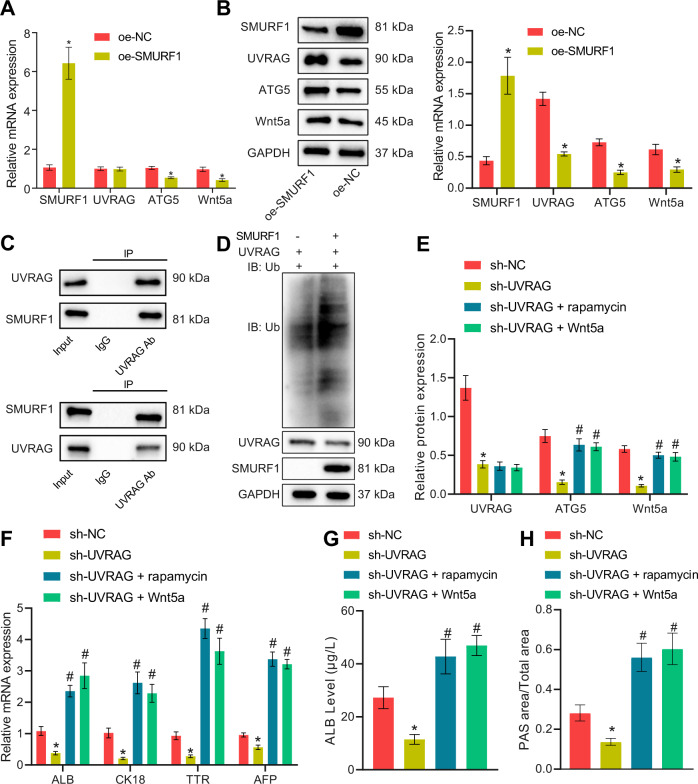
SMURF1 promotes ubiquitination of UVRAG and regulates ATG5/Wnt5a to suppress HLC differentiation of mouse BMSCs. **A** RT-qPCR to determine the expression of UVRAG, ATG5, and Wnt5a after overexpressing SMURF1. **B** The expression of UVRAG, ATG5, and Wnt5a after overexpression of SMURF1 detected by Western blot analysis. **C** The binding between SMURF1 and UVRAG assessed by Co-IP analysis. **D** The effect of SMURF1 on the ubiquitination of UVRAG measured by Co-IP analysis. **E** The effect of concomitant treatment of rapamycin or recombinant Wnt5a and sh-UVRAG on the expression of ATG5, Wnt5a assessed by Western blot analysis. **F** RT-qPCR to determine the expression of hepatocyte-related genes in BMSCs after 14 days of simultaneous treatment of rapamycin or recombinant Wnt5a and sh-UVRAG. **G** ELISA assay to detect the ALB content in culture supernatant of BMSCs after 14 days of concomitant treatment of rapamycin or recombinant Wnt5a and sh-UVRAG. **H** PAS staining to detect the accumulation of glycogen in BMSCs after 14 days of simultaneous treatment of rapamycin or recombinant Wnt5a and sh-UVRAG. *p < 0.05 *vs*. BMSCs transfected with oe-NC/sh-NC; ^#^*p* < 0.05 *vs*. BMSCs transfected with sh-UVRAG. The cell experiments were repeated three times.

Finally, the downstream mechanism of SMURF1 in cirrhosis was explored. In response to SMURF1 restoration in BMSCs, UVRAG mRNA expression showed no obvious changes whereas a reduction was noted in UVRAG protein expression; accompanied by reduced mRNA and protein expression of ATG5 and Wnt5a (Fig. 8A, B). Since SMURF1 is a ubiquitin ligase, the binding between SMURF1 and UVRAG was determined with Co-immunoprecipitation (Co-IP), which demonstrated that there was binding between SMURF1 and UVRAG (Fig. 8C). Furthermore, the ubiquitin-binding of UVRAG was measured after co-transfection of SMURF1 and UVRAG, and the results manifested that overexpression of SMURF1 promoted the ubiquitination of UVRAG, thus mediated its degradation (Fig. 8D). In order to clarify the axis mediatory relationship among UVRAG/ATG5/Wnt5a, the autophagy activator rapamycin or recombinant Wnt5a was applied when UVRAG was silenced. The results documented that ATG5 and Wnt5a expression was markedly decreased after UVRAG silencing, which was annulled by treatment with rapamycin or recombinant Wnt5a, and there was a positive feedback mechanism between ATG5 and Wnt5a (Fig. 8E). Moreover, UVRAG silencing reduced hepatocyte-related gene expression, ALB secretion, and glycogen content in the BMSCs, whereas rapamycin or recombinant Wnt5a counteracted this effect (Fig. 8F–H).

**Fig. 9:**
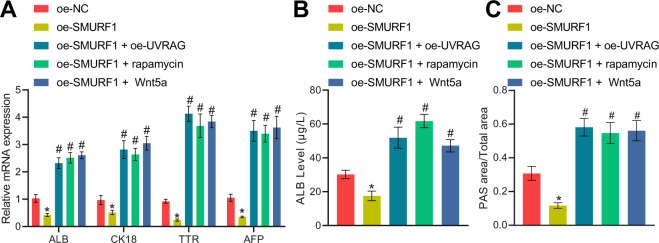
SMURF1/UVRAG/ATG5/Wnt5a axis affects cirrhosis in mice. **A** RT-qPCR to detect the expression of hepatocyte-related genes in BMSCs after simultaneous treatment of oe-SMURF1 and oe-UVRAG, rapamycin, or recombinant Wnt5a for 14 days. **B** ELISA to detect the secretion of ALB by BMSCs after simultaneous treatment of oe-SMURF1 and oe-UVRAG, rapamycin, or recombinant Wnt5a for 14 days. **C** PAS staining of glycogen accumulation in BMSCs after simultaneous treatment of oe-SMURF1 and oe-UVRAG, rapamycin, or recombinant Wnt5a for 14 days. *p < 0.05 *vs*. BMSCs transfected with oe-NC; ^#^*p* < 0.05 *vs*. BMSCs transfected with oe-SMURF1. The cell experiments were repeated three times.

To further identify whether SMURF1 assumed a role in affecting HLC differentiation from BMSCs through the UVRAG/ATG5/Wnt5a axis, in vitro and in vivo experiments were conducted. Oe-UVRAG, rapamycin, or recombinant Wnt5a was utilized in BMSCs while overexpressing SMURF1. Overexpression of SMURF1 curtailed hepatocyte-related gene expression, ALB secretion, and glycogen content in BMSCs, which was negated by oe-UVRAG, rapamycin, or recombinant Wnt5a (Fig. 9A–C). Further, we testified the findings in the cirrhosis mouse model and obtained similar results as in vitro experiments: injection of SMURF1 overexpression HGF-induced BMSCs aggravated the degree of cirrhosis in mice, which was abolished by oe-UVRAG, rapamycin, or recombinant Wnt5a (Supplementary Table 4).

To sum up, SMURF1 repressed HLC differentiation of BMSCs and cirrhosis of mice via UVRAG/ATG5/Wnt5a axis.

## Discussion

Cirrhosis is a diffuse hepatic process with the features of fibrosis and structurally abnormal nodules, which represents the ultimate histological change of numerous chronic liver diseases [23]. As reported, lncRNAs has been implicated in liver fibrosis, which can be utilized as biomarkers of liver fibrosis [24]. However, there exists limited evidence in regard to the role of lncRNAs in cirrhosis. Therefore, our work was designed to probe whether lncRNA SNHG1 influenced cirrhosis and the potential mechanism, and thus illustrated that silencing of lncRNA SNHG1 might promote HLC differentiation of BMSCs to protect against cirrhosis by activating UVRAG/ATG5/Wnt5a axis via downregulation of miR-15a-targeted SMURF1.

Our work identified that lncRNA SNHG1 downregulation accelerated HLC differentiation of BMSCs by upregulating ALB, CK18, TTR, and AFP and increasing glycogen content. It has been documented that ALB is one of the hepatocyte markers [25], and that HLCs possess some functional hepatic activity because these cells secrete urea, alpha-1-antitrypsin, and ALB [26]. Besides, TTR, AFP, and CK18 are widely recognized as hepatocyte-specific genes [27, 28]. Moreover, a prior work indicated that ALB and CK18 expression and glycogen content were elevated after human BMSCs were induced to differentiate HLCs by HGF [29]. Partially consistent with our results, the existing studies illustrated that lncRNA SNHG1 overexpression was capable of reducing BMSC differentiation [12, 30]. Therefore, we could conclude that lncRNA SNHG1 might repress HLC differentiation of BMSCs.

Moreover, in our work, injection of HGF-induced BMSCs, especially HGF-induced BMSCs silencing lncRNA SNHG1, diminished liver fibrosis to repress cirrhosis in vivo. Liver fibrosis has been recognized as a major lesion of the liver that results in cirrhosis at the end stage [31]. Currently, decreasing fibrosis is one of the standards for the treatment of cirrhosis [32]. Moreover, BMSCs may differentiate into organ parenchymal cells to treat cirrhosis [33]. Specifically, it was revealed in a prior work that the differentiation of BMSCs into HLCs was able to alleviate cirrhosis [34]. More importantly, BMSCs have emerged as a therapy for liver fibrosis [35]. It was noted in the study of Ma et al. that fibrotic area was obviously reduced by BMSCs treatment in model animals with CCl4-induced liver fibrosis [32]. Besides, a prior work elucidated that ectopically expressed lncRNA SNHG1 could accelerate the progression of liver cancer, for which cirrhosis is a significant risk factor [36]. Therefore, lncRNA SNHG1 silencing might be involved in the protective role of BMSCs against cirrhosis.

As reported, lncRNA SNHG1 bound to miR-15a to orchestrate cardiomyocytes hypertrophy [13]. Consistently, our work found that lncRNA SNHG1 repressed HLC differentiation of BMSCs in vitro and augmented liver fibrosis in mice with cirrhosis by binding to miR-15a. miR-15a has been noted to show high expression during hepatogenic differentiation of MSCs induced by HGF [21], suggesting that miR-15a overexpression might accelerate HLC differentiation from BMSCs. In addition, miR-15a was observed to be associated with the mediation of liver fibrosis in a gerbil model of fatty liver fibrosis treated with exenatide [21]. Moreover, our data revealed that miR-15a targeted SMURF1, which enhanced ubiquitination of UVRAG, to promote BMSCs to differentiate to HLC in vitro and repress liver fibrosis in mice with cirrhosis. Partially concordant with our results, SMURF1 repressed BMSC proliferation and differentiation [37]. A previous study illustrated that SMURF1 upregulation caused an increase of high glucose (HG)-induced renal fibrosis in glomerular mesangial cells and diabetic mice kidneys [38]. Furthermore, SMURF1 resulted in ubiquitination of UVRAG in HCC cells [17]. Another critical finding in our study was that overexpressed UVRAG promoted ATG5/Wnt5a activation to decrease HLC differentiation of BMSCs, thus alleviating cirrhosis. It is well-known that UVRAG and ATG5 are both autophagy-related genes [39]. ATG5 was able to manipulate a positive feedback loop between Wnt5a and autophagy in melanoma cells [19]. In addition, ATG5 expression was elevated after liver fibrosis was alleviated [40]. Moreover, a prior study elaborated that Wnt5a could promote HLC differentiation of MSCs [41].

**Fig. 10:**
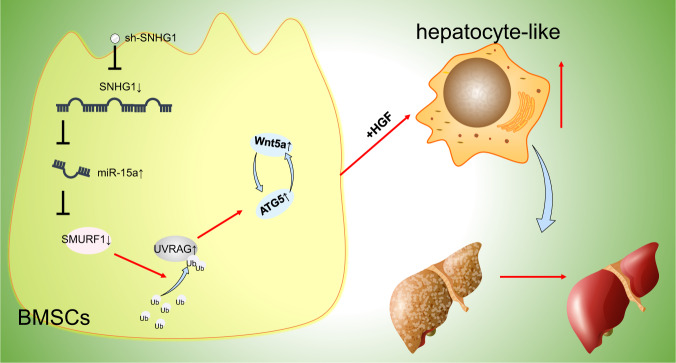
Mechanism graph of lncRNA SNHG1 modulating the miR-15a/SMURF1/UVRAG/ATG5/Wnt5a axis in HLC differentiation of BMSCs and cirrhosis. shRNA-mediated lncRNA SNHG1 silencing promotes HLC differentiation of BMSCs to alleviate cirrhosis by activating UVRAG/ATG5/Wnt5a axis via downregulation of miR-15a-targeted SMURF1.

In summary, our findings supported the repressive effect of lncRNA SNHG1 silencing in cirrhosis. Briefly, lncRNA SNHG1 silencing was observed to downregulate SMURF1 by upregulating miR-15a, diminishing ubiquitination of UVRAG to activate ATG5/Wnt5a axis, which accelerated HLC differentiation from BMSCs and repressed liver fibrosis to inhibit cirrhosis (Fig. 10). This finding adds to our understanding of the complex mechanism of lncRNA SNHG1/miR-15a/SMURF1/UVRAG/ATG5/Wnt5a axis in cirrhosis progression and provides a potential new therapeutic target for cirrhosis prevention. However, studies should be performed to warrant further exploration in the clinical setting.

## Methods

### Protocols

In this study, mouse cirrhosis models were treated with BMSCs derived from lncRNA SNHG1-silenced mice. By measuring the HLC differentiation of mouse MSCs in model mice, the expression of hepatocyte-related genes in liver tissues (ALB, CK18, TTR, and AFP), the secretion of serum albumin, glycogen synthesis, and the number of hepatic pseudolobules and collagen fibers, we evaluated the therapeutic effect of mouse BMSCs on cirrhosis.

### Isolation and incubation of BMSCs

Femurs were collected from BALB/c mice and rinsed with α-MEM medium supplemented with 10% fetal bovine serum (FBS, ICN Biochemicals, Costa Mesa, CA, USA), 100 U/mL penicillin G, and 100 μg/mL streptomycin (Nacalai Tesque, Kyoto, Japan) for several times, after which bone marrow was harvested and centrifuged at 1500 rpm for 5 min. Pelleted cells were plated, and 4 h after cell attachment to the surface, the supernatant (containing non-adherent cells) was discarded. Cells were supplemented with fresh α-MEM medium for 3 days incubation, and then trypsinized to obtained purer BMSCs. Then, BMSCs were passaged and seeded at 10,000–12,000 cells/cm^2^, and BMSCs at 4th passage were adopted for following use.

Mouse normal hepatocytes NCTC 1469 (Wuhan Procell Life Science & Technology, Wuhan, Hubei, China) were cultured using Dulbecco’s Modified Eagle Medium with 10% FBS. Both BMSCs and NCTC 1469 cells were incubated in a 37 °C and 5% CO_2_ incubator.

In addition, the specific drugs added in some experiments were: hepatocyte growth factor (HGF; 20 μg/mL, Sigma-Aldrich, St Louis, MO, USA), rapamycin (1 μM, MCE, USA), and recombinant Wnt5a (0.2 μg/mL, Abcam, Cambridge, UK).

### Identification of BMSCs immunophenotype by flow cytometry

Flow cytometry was performed to detect the expression of BMSC surface antigen markers o CD105 (an MSC marker), CD34 (to exclude primary hematopoietic and endothelial cells), and CD45 (to exclude leukocytes). BMSCs were trypsinized and incubated with the following antibodies for 30 min: anti-CD105 (MCA1557F; Bio-Rad, Hercules, CA, USA), anti-CD34 (MBS438077; MyBioSource, San Diego, CA, USA), and anti-CD45 (ab10558; Abcam). A FAC Scan flow cytometer (Becton Dickinson, Heidelberg, Germany) was employed, and flow cytometric data were analyzed with the FlowJo software (Tree Star Inc., Ashland, OR, USA).

### Immunofluorescence staining

BMSCs were seeded in 24-well plates, and the BMSC marker CD105 and hepatocyte markers AFP and ALB were analyzed with immunofluorescence staining. Briefly, cells were fixed with 4% PFA for 30 min, washed, and blocked with 1% BSA for 30 min, followed by incubation with primary antibodies against CD105 (ab221675, 1:2000, Abcam, UK), ALB (ab222923, 1:2000, Abcam) and AFP (ab213328, 1:2000, Abcam) overnight. Afterward, the cells were incubated with fluorescence-conjugated secondary antibodies for 2 h and subsequently with DAPI (OriGene Technologies, Rockville, MD, USA) for 20 min. Stained cells were then observed with a fluorescence microscope (Olympus, Hamburg, Germany).

### Cell transfection

Logarithmically growing BMSCs were trypsinized and seeded in 6-well plates at an appropriate cell density for reaching 70% confluence on the following day of transfection. Cell transfection was implemented as per the manuals of Lipofectamine 3000 (Invitrogen, Carlsbad, CA, USA) with the medium being replaced with a fresh medium 6–8 h after transfection. The subsequent experiments were performed after continuing to culture cells for 24–48 h.

BMSCs were transfected with short hairpin RNA (sh)-negative control (NC) or sh-SNHG1 (#1, #2, and #3) to investigate the effect of lncRNA SNHG1 on HLC differentiation of BMSCs and expression of downstream related factors. BMSCs were transfected with overexpression (oe)-NC, oe-SMURF1, NC mimic, or miR-15a mimic to study the impact of miR-15a and SMURF1 on HLC differentiation of BMSCs. To observe the influence of lncRNA SNHG1/miR-15a/SMURF1 axis on HLC differentiation of BMSCs, BMSCs were transfected with sh-NC, sh-SNHG1, NC mimic, miR-15a mimic, inhibitor NC, miR-15a inhibitor, oe-NC, or oe-SMURF1. To assess the effect of SMURF1 on UVRAG/ATG5/Wnt5a axis, BMSCs were transfected with oe-NC, oe-SMURF1, oe-UVRAG, sh-NC, sh-UVRAG, or oe-Ubiquitination (Ub). Overexpression plasmids were constructed using pCDNA3.1 vector, and the silencing plasmids were constructed using pLKO.1 vector (both from GenePharma, Shanghai, China).

Cells transfected with the silencing plasmids were selected with 1 μg/mL puromycin (MCE), and cells transfected with overexpression plasmids were screened using 1 μg/mL neomycin (MCE).

### RT-qPCR

Subsequent to the isolation of total RNA of cells and tissues using TRIzol (Invitrogen), a miRcute Plus miRNA First-Strand cDNA Synthesis Kit (TIANGEN, Beijing, China) was applied to reversely transcribed RNA of miR-15a, and a Prime ScriptTM RT Kit (Takara, Tokyo, Japan) to reversely transcribed RNA of other genes. RT-qPCR was implemented using a TransStart Tip Green qPCR SuperMix (TransGen Biotech, Beijing, China) with U6 (for miR-15a) and glyceraldehyde-3-phosphate dehydrogenase (GAPDH, for other genes) as normalizers. The relative gene expression was evaluated using the 2^−ΔΔCT^ method. The primers are listed in Supplementary Table 5.

### ELISA

The cell culture supernatant was harvested and centrifuged at 3000 rpm for 10 min to remove particles and polymers. The enzyme-linked reaction was conducted using an ALB ELISA Assay Kit (DUMA, Shanghai, China) and the content of ALB in the cell supernatant was quantified by a microplate reader.

### PAS staining

Cells were stained using a Glycogen Staining Kit (G1360, Solarbio, Beijing, China). Glycogen accumulation was monitored microscopically.

### Establishment of cirrhosis mouse model

BALB/c mice (Vital River Laboratories, Beijing, China) aged 6–8 weeks were housed in a specific pathogen-free area at 18–22 °C with 40–70% humidity and fed with regular diet with free access to food. After a week of acclimatization, mice in good health were injected intraperitoneally with carbon tetrachloride (CCl4) at a dose of 1 mL/kg body weight twice a week for 4 weeks. Phenobarbital was added into drinking water at a concentration of 0.25 g/L to enhance liver injury and establish a mouse model of cirrhosis. During the modeling period, the status, survival, and weight of mice were recorded every day. Four weeks after the administration, the liver of mice was attained to observe the nodular condition of the liver surface or to undergo HE staining and Masson’s collagen fiber staining to evaluate the success of the modeling.

### Intravenous injection of BMSCs for treatment of cirrhosis in mice

Logarithmically growing BMSCs were trypsinized and the cell density was adjusted into 1 × 10^4^ cells/μL. The 100 μL cell suspension was supplemented with 4 μg plasmids to transfect cells. After the efficiency was verified, amplification and screening were carried out in vitro. After more than 30 days of culture, cirrhotic mice were treated by tail vein injection of BMSCs (200 μL and 1 × 10^6^ cells/time) once a week for 4 weeks (synchronous with 4 weeks of modeling). Four weeks after the end of treatment, the liver of mice was obtained to assess the therapeutic effect (gross view, HE staining, and Masson’s collagen fiber staining). During the treatment, the status, survival, and weight of the mice were recorded every day.

### HE staining

After the mice were euthanized, the liver tissue was harvested to observe the nodules on the surface of the liver by naked eyes and photographed. After 10% formaldehyde fixation, the liver was paraffin-embedded, sliced, and stained successively with hematoxylin and eosin according to the protocols of a HE staining kit (G1120, Solarbio). After neutral resin mounting, the formation of pseudolobules in liver slices was observed under a microscope.

### Masson’s collagen fiber staining

Following euthanasia of the mice, the nodules on the surface of liver were observed by naked eyes. Subsequent to 10% formaldehyde fixation, the liver was paraffin-embedded and sectioned for Masson’s (G1340, Solarbio) collagen fiber staining. After sealing with neutral resin, liver fibrosis was observed under the microscope.

### Western blot analysis

The cultured cells were harvested by trypsin digestion and lysed with enhanced Radio-Immunoprecipitation assay cell lysis buffer encompassing protease inhibitor (BOSTER, Wuhan, Hubei, China). The protein concentration was estimated by a bicinchoninic acid protein quantitative Kit (BOSTER). The proteins were separated by sodium dodecyl sulfate polyacrylamide gel electrophoresis and electroblotted to a polyvinylidene fluoride membrane that was sealed at room temperature for 2 h with 5% bovine serum albumin to block nonspecific binding. Overnight probing was implemented with primary antibodies (1:1000, Cell Signaling Technology, Beverly, MA, USA) to SMURF1 (#2714), GAPDH (#5174), UVRAG (#13115), ATG5 (#12994), Wnt5a (#2392), and Ub (#5174) at 4 °C, followed by 1 h reprobing with horseradish peroxidase-tagged goat anti-rabbit IgG (ab205719, 1:2000, Abcam) at room temperature. Then the membrane was incubated for 1 min with electrogenerated chemiluminescence (ECL) working solution (EMD Millipore Corp., Billerica, MA, USA). Subsequent to discarding of excess ECL reagent, the membrane was sealed with plastic wrap, and exposed with X-ray film in the dark box for 5–10 min before development and fixation. The image J software was adopted for gray value quantitative analysis of protein bands with GAPDH as a normalizer.

### Co-IP

Binding between UVRAG and SMURF1 was assessed: the medium was discarded, and cells were washed with phosphate buffer saline (PBS) two times with the removal of PBS. The 1 mL IP lysis buffer containing protease inhibitors (BOSTER) was supplemented to cells and left on ice for 15 min. Following 10 min cell centrifugation at 12,000 rpm, the supernatant was collected into a new centrifuge tube. Cell lysates, anti-UVRAG antibody/anti-SMURF1 antibody, and 25 μL protein A sepharose were mixed completely before overnight incubation at 4 °C on a vertical shaker. Magnetic beads were centrifuged at 1000 rpm and 4 °C for 5 min with the supernatant discarded and washed three times with IP lysis buffer. Magnetic beads were supplemented to the same volume of 2 × SDS loading buffer, gently mixed, and heated at 95 °C for 10 min. The supernatant was attained for Western blot analysis to detect the expression of SMURF1 and UVRAG, and 10% of the supernatant simultaneously for Input detection.

Detection of the ubiquitination level of UVRAG: the medium was discarded, and cells were washed with phosphate buffer saline (PBS) two times with the removal of PBS. The 1 mL IP lysis buffer containing protease inhibitors (BOSTER) was supplemented to cells and left on ice for 15 min. Following 10 min cell centrifugation at 12000 rpm, the supernatant was collected into a new centrifuge tube. Cell lysates, anti-UVRAG antibody, and 25 μL protein A sepharose were mixed completely before overnight incubation at 4 °C on a vertical shaker. Magnetic beads were centrifuged at 1000 rpm and 4 °C for 5 min with the supernatant discarded, and washed three times with IP lysis buffer. Magnetic beads were supplemented to the same volume of 2 × SDS loading buffer, gently mixed, and heated at 95 °C for 10 min. The supernatant was attained for Western blot analysis to detect the ubiquitination level of UVRAG, and 10% of the supernatant simultaneously for Input detection.

### Dual-luciferase reporter assay

The binding sites and sequences of miR-15a to lncRNA SNHG1 and SMURF1 were predicted by Target Scan, a target prediction website. LncRNA SNHG1 wild-type sequence (lncRNA SNHG1-W), lncRNA SNHG1 mutant sequence (lncRNA SNHG1-M), SMURF1 3' untranslated region (UTR) wild-type sequence (SMURF1 3'UTR-W), and SMURF1 3'UTR mutant sequence (SMURF1 3'UTR-M) were constructed with the sequence of 200 bp upstream and downstream of this site. The above sequences (GenePharma) were recombined with the dual-luciferase reporter system pmir GLO vector. The recombinant vectors were identified by PCR and gene sequencing to demonstrate successful recombinant vector construction. miR-15a mimic or mimic NC (GenePharma) were transfected with the above sequences into 293 T cells (purchased from American Type Culture Collection [Rockville, MD, USA] and incubated in DMEM medium supplemented with 10% FBS and 1% antibiotics at 37 °C, 5% CO_2_) and, 48 h later, luciferase activity was measured using the dual-luciferase Kit (Promega, Madison, WI, USA) and calculated by the ratio of firefly luciferase/Renilla luciferase.

### Statistical analysis

SPSS 22.0 was employed for statistical analysis. The measurement results were summarized as mean ± standard deviation. The tests conformed to normal distribution and homogeneity of variance. Unpaired *t*-test was adopted for comparison between the two groups. One-way analysis of variance (ANOVA) or repeated measurement ANOVA was implemented for comparison among multiple groups. *p* < 0.05 was considered to be statistically obvious difference

## Supplementary information

Supplementary Figure 1

Supplementary Tables

## Data Availability

The datasets generated/analyzed during the current study are available from the corresponding author upon reasonable request.
