# Supplementary material for: Long noncoding RNA SNHG1 silencing accelerates hepatocyte-like cell differentiation of bone marrow-derived mesenchymal stem cells to alleviate cirrhosis via the microRNA-15a/SMURF1/UVRAG axis
Source: Cell Death Discov. 2022 Feb 22;8:77. doi: 10.1038/s41420-022-00850-8 (PMC8863836; doi:10.1038/s41420-022-00850-8)
Supplement: Supplementary file 1 — Supplementary Figure 1 [file 41420_2022_850_MOESM1_ESM.docx]

**
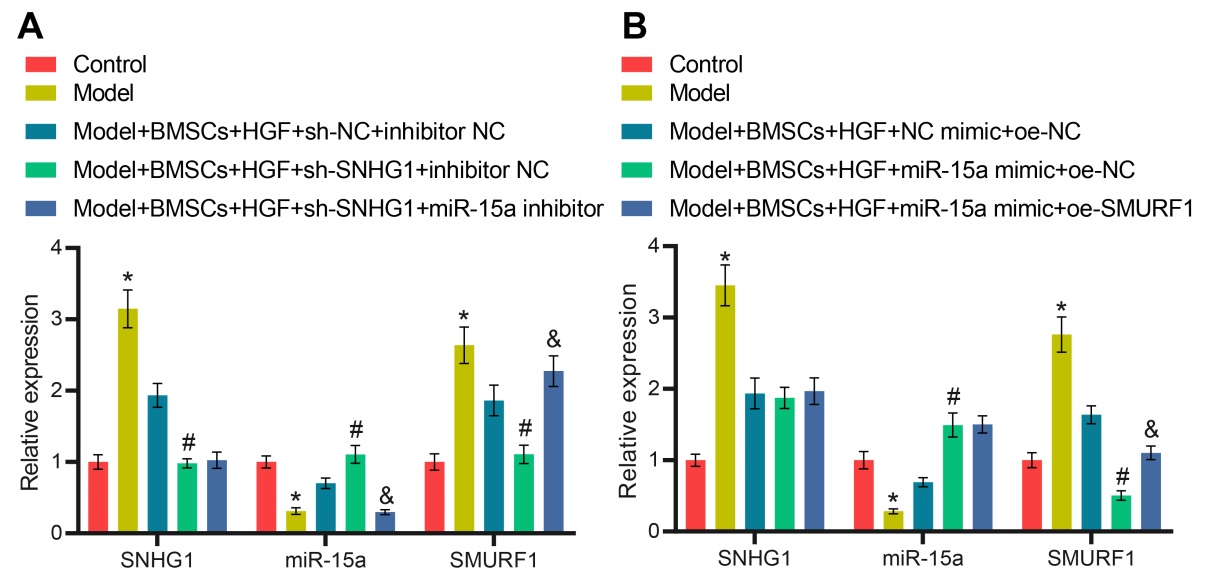
**

**Supplementary Fig. 1 The expression of lncRNA SNHG1, miR-15a and SMURF1 in mouse livers determined with qRT-PCR.** A, The expression of lncRNA SNHG1, miR-15a and SMURF1 in livers of control mice, cirrhotic mice, and cirrhotic mice treated with HGF-stimulated BMSCs silencing lncRNA SNHG1 alone or in combination with miR-15a. B, The expression of lncRNA SNHG1, miR-15a and SMURF1 in livers of control mice, cirrhotic mice, and cirrhotic mice treated with HGF-stimulated BMSCs overexpressing miR-15a alone or in combination with SMURF1. * *p* < 0.05 *vs.* Control mice; # *p* < 0.05 *vs.* the Model + BMSCs + HGF + sh-NC + inhibitor NC or the Model + BMSCs + HGF + mimic-NC + oe-NC group; & *p* < 0.05 *vs.* the Model + BMSCs + HGF + sh-SNHG1 + inhibitor NC or the Model + BMSCs + HGF + miR-15a mimic + oe-NC group. N = 6.
