# Supplementary material for: Long noncoding RNA SNHG1 silencing accelerates hepatocyte-like cell differentiation of bone marrow-derived mesenchymal stem cells to alleviate cirrhosis via the microRNA-15a/SMURF1/UVRAG axis
Source: Cell Death Discov. 2022 Feb 22;8:77. doi: 10.1038/s41420-022-00850-8 (PMC8863836; doi:10.1038/s41420-022-00850-8)
Supplement: Supplementary file 2 — Supplementary Tables [file 41420_2022_850_MOESM2_ESM.docx]

**Supplementary Table 1** Collagen fibers of normal mice, cirrhotic mice and treated mice

| **Groups** | **Collagen fibers** |
| --- | --- |
| Control | 0 |
| Model | 18.61±2.95* |
| Model + BMSC | 20.67±3.10 |
| Model + BMSC + HGF | 7.56±1.19# |
| Model + BMSC + HGF + sh-SNHG1 | 4.06±0.70& |

* *p* < 0.05 *vs.* Control (control mice); # *p* < 0.05 *vs.* Model (cirrhotic mice); & *p* < 0.05 *vs.* Model + BMSC + HGF (cirrhotic mice injected HGF-induced BMSCs). N = 6 mice/group. BMSC, bone marrow-derived mesenchymal stem cell.

**Supplementary Table 2** The effect of lncRNA SNHG1/miR-15a axis on collagen fibers of mice

| Groups | Collagen fibers |
| --- | --- |
| Control | 0 |
| Model | 18.61 ± 2.95 * |
| Model + BMSC + HGF + sh-NC + inhibitor NC | 6.78 ± 0.72 |
| Model + BMSC + HGF + sh-SNHG1 + inhibitor NC | 0.17 ± 0.28 # |
| Model + BMSC + HGF + sh-SNHG1 + miR-15a inhibitor | 17.11 ± 1.60& |

* *p* < 0.05 *vs.* Control (control mice); # *p* < 0.05 *vs.* Model + BMSC + HGF + sh-NC + inhibitor NC (cirrhotic mice treated with BMSC + HGF + sh-NC + inhibitor NC); & *p* < 0.05 *vs.* Model + BMSC + HGF + sh-SNHG1 + inhibitor NC (cirrhotic mice treated with BMSC + HGF + sh-SNHG1 + inhibitor NC). N = 6 mice/group. BMSC, bone marrow-derived mesenchymal stem cell.

**Supplementary Table 3** The effect of miR-15a/SMURF1 axis on collagen fibers of mice

| Groups | Collagen fibers |
| --- | --- |
| Control | 0 |
| Model | 18.61 ± 2.95* |
| Model + BMSC + HGF + NC mimic + oe-NC | 7.06 ± 0.49 |
| Model + BMSC + HGF + miR-15a mimic + oe-NC | 0.28 ± 0.33# |
| Model + BMSC + HGF + miR-15a mimic + oe-SMURF1 | 17.78 ± 1.33& |

* *p* < 0.05 *vs.* Control (control mice); # *p* < 0.05 *vs.* Model + BMSC + HGF + NC mimic + oe-NC (cirrhotic mice treated with BMSC + HGF + NC mimic + oe-NC); & *p* < 0.05 *vs.* Model + BMSC + HGF + miR-15a mimic + oe-NC (cirrhotic mice treated with BMSC + HGF + miR-15a mimic + oe-NC). N = 6 mice/group. BMSC, bone marrow-derived mesenchymal stem cell.

**Supplementary Table 4** The effect of SMURF1/UVRAG/ATG5/Wnt5A axis on collagen fibers of mice

| Groups | Collagen fibers |
| --- | --- |
| Model + BMSC + HGF + oe-NC | 6.61 ± 1.36 |
| Model + BMSC + HGF + oe-SMURF1 | 18.78 ± 2.54* |
| Model + BMSC + HGF + oe-SMURF1 + oe-UVRAG | 0.50 ± 0.46# |
| Model + BMSC + HGF + oe-SMURF1 + rapamycin | 0.17 ± 0.18# |
| Model + BMSC + HGF + oe-SMURF1 + Wnt5a | 0.28 ± 0.25# |

* *p* < 0.05 *vs.* Model + BMSC + HGF + oe-NC (cirrhotic mice treated with BMSC + HGF + oe-NC); # *p* < 0.05 *vs.* Model + BMSC + HGF + oe-SMURF1 (cirrhotic mice treated with BMSC + HGF + oe-SMURF1). N = 6 mice/group. BMSC, bone marrow-derived mesenchymal stem cell.

**Supplementary Table 5** Primers for RT-qPCR

| Gene | Sequence (5' -3') |
| --- | --- |
| GAPDH | Forward: TGGATTTGGACGCATTGGTC |
|  | Reverse: TTTGCACTGGTACGTGTTGAT |
| ALB | Forward: CAAGAGTGAGATCGCCCATCG |
|  | Reverse: TTACTTCCTGCACTAATTTGGCA |
| CK18 | Forward: CAGCCAGCGTCTATGCAGG |
|  | Reverse: CCTTCTCGGTCTGGATTCCAC |
| TTR | Forward: CTGCTGTAGACGTGGCTGTAA |
|  | Reverse: CTTCCAGTACGATTTGGTGTCC |
| AFP | Forward: AGCTTCCACGTTAGATTCCTCC |
|  | Reverse: ACAAACTGGGTAAAGGTGATGG |
| miR-15a | Forward: TAGCAGCACATAATGGTTTGTG |
|  | Reverse: Universal revers primer |
| U6 | Forward: CTCGCTTCGGCAGCACA |
|  | Reverse: Universal revers primer |
| SMURF1 | Forward: AGCATCAAGATCCGTCTGACA |
|  | Reverse: CCAGAGCCGTCCACAACAAT |
| LncRNA SNHG1 | Forward: CCCAGGATGAGTGCAGGTTT |
|  | Reverse: TCCTTCACACGCAGCTCATT |

**Note:** ALB, albumin; AFP, alpha-fetoprotein; CK18, cytokeratin 18; GAPDH, glyceraldehyde-3-phosphate dehydrogenase; miR-15a, microRNA-15a; SMURF1, Smad ubiquitin regulatory factor 1; TTR, transthyretin
